# Accuracy of intra ocular lens calculation formulae in patients with pseudoexfoliation syndrome

**DOI:** 10.1007/s10792-024-03195-6

**Published:** 2024-06-24

**Authors:** Inbal Gazit, Anna Gershevich, Adi Einan-Lifshitz, Eran Pras, Graham D. Barrett, Lior Or

**Affiliations:** 1 Department of Ophthalmology, Shamir Medical Center, Be’er Ya’acov, Israel; 2 Sackler Faculty of Medicine, Tel Aviv University, Tel Aviv, Israel; 3 Lions Eye Institute, Perth, WA Australia; 4 Centre for Ophthalmology and Visual Science, University of Western Australia, Perth, WA Australia

**Keywords:** Cataract, Intraocular lens calculation, Pseudoexfoliation syndrome

## Abstract

**Background:**

The purpose of this study was to investigate the visual and refractive outcomes in patients with pseudoexfoliation (PXF) undergoing routine cataract surgery and to compare the accuracy of intraocular lens (IOL) power calculation formulae.

**Methods:**

Retrospective case-series study from Shamir medical center, a public hospital, Israel. Medical records of patients who underwent routine cataract surgery between January 2019 and August 2021 were investigated. Postoperative visual acuity and manifest refraction were examined. The error in predicted refraction and IOL power calculation accuracy within a range of ± 0.50 to ± 1.00 diopters were compared between different IOL calculating formulae.

**Results:**

151 eyes of 151 patients ages 73.9 ± 7.1 years were included in this study- 58 eyes in the PXF group and 93 eyes in the control group. The mean absolute error (MAE) for the BUII formula was 0.63D ± 0.87 for the PXF group and 0.36D ± 0.48 for the control group (*p* < 0.05). The MAE for the Hill-RBF 3.0 formula was 0.61D ± 0.84 for the PXF group and 0.42D ± 0.55 for the control group (*p* = 0.05). There were significant differences in MAE and MedAE between PXF group and control group measures (*p* < 0.05). In the PXF group there were no significant differences between the different formulae.

**Conclusions:**

There were significant differences in accuracy of IOL power calculations in all formulae between PXF group and control group measures. PXF patients show hyperopic shift from predicted refraction. Barret universal II formula had the highest proportion of eyes with absolute error in prediction below or equal to 0.50 D in both PXF and control groups.

## Introduction

First described in 1971, pseudoexfoliation (PXF) is an age-related syndrome affecting the anterior segment of the eye, along with other organs in the body. [1–4] PXF is known to be associated with the development of cataract [5], ocular hypertension and open angle glaucoma [6].

In the context of cataract surgery, PXF is associated with intra and postoperative complications [7]. The intra-operative complications include small pupil, shallow or hyper-deep anterior chamber, zonule instability and dialysis, capsule fragility, posterior capsule tear and dropped nucleus. Post-operative complications include intra-ocular pressure spike, corneal edema, aqueous flare, posterior synechias, cystoid macular edema, anterior capsule contraction (phimosis), posterior capsular opacification, and IOL subluxation or dislocation [7, 8].

Maximal accuracy of IOL calculations methods preceding cataract surgery is nowadays the standard of care, since modern cataract surgery is expected to be both a rehabilitative and a refractive surgery. The accuracy of IOL calculations is dependent on the pre-operative biometry measurements including axial length, keratometry readings, anterior chamber depth, white-to-white as well as IOL constants.

A Recent small study demonstrated refractive outcomes post cataract surgery are significantly worse in patients with PXF compared to controls. The study examined only three IOL calculation formulae: Barrett Universal II (BUII), Hill-RBF 3.0 and SRK/T and found no difference between the formulae [9].

The aim of this study was to evaluate the post-operative refractive outcomes of patients with PXF, to compare them to the outcomes in matched patients without PXF, and to determine which of the most used IOL calculation formulae (Barrett Universal II (BUII), Haigis, Hoffer Q, Holladay I, Hill-RBF 3.0 and SRK/T) is the most accurate in patients with PXF.

## Patients and methods

### Study design

Medical records of patients with PXF, who underwent cataract surgery between January 2019 and August 2021 with Acrysof IOL (SA60AT, Alcon Laboratories, TX, USA) by two different surgeons (A.E.L and E.P.) were retrospectively analysed as the PXF group. PXF group included patients with PXF syndrome with or without PXF glaucoma.

Medical records of consecutive patients, who underwent uncomplicated cataract surgery at the same time frame, using the same IOL by the same two surgeons were retrospectively analysed as the control group. All patients underwent pre-operative ocular biometry analysis using the IOL Master 700 (Carl Zeiss Meditec AG, Jena, Germany). All patients underwent a standard cataract surgery with local anesthesia through a 2.4 incision in 90 degrees. Patients with keratoconus, a history of refractive surgery, retinal disease, previous glaucoma related surgery or any other condition influencing visual activity and any deviation from standard surgery course were excluded.

IOL was chosen pre-operatively based on BUII formula. The target spherical equivalent was the closest to plano.

Predicted postoperative refraction (PPOR) for spherical outcome was calculated retrospectively for both groups with the Barrett Universal II (BUII), Haigis, Hoffer Q, Holladay I, Hill-RBF 3.0 and SRK/T. The BUII, Haigis, Holladay I, Hoffer Q and SRK/T formulae were calculated using IOL Master 700 software and Hill-RBF 3.0 was calculated by using the online calculator [10].

The error in predicted spherical equivalent (SE) was calculated for each formula by subtracting the PPOR from the subjective refraction. The IOL constant was optimised for each formula, to reduce the mean error (ME) to approximately zero (< 0.01) [11]. Mean absolute error (MAE), median absolute error (MedAE), standard deviation (SD) and the percentage of eyes in each subgroup within ± 0.50, ± 0.75 and ± 1.00 D of target were calculated.

Patients were examined 1 day, 1 week, and 4–6 weeks after surgery. Slitlamp examination was conducted at every visit. Subjective refraction was performed by a single trained optometrist at least 4 weeks postoperatively, using the cross-cylinder technique at 6 m. Patients with post-operative BCVA of 6/12 or worse (0.3 LogMar or worse) were excluded from the study in order to ensure subjective refraction is kept as reliable as possible.

### Primary and secondary outcomes

The proportion of eyes within absolute prediction error (PE) was the primary outcome. MAE and MedAE are secondary outcomes.

### Sample size

We designed the study to have at least 80% power and a significance level of 5%. Given an expected postoperative MAE of 0.3 among control group and postoperative MAE of 0.5 among PXF patients with a standard deviation of 0.3 [12]. Exposed to non-exposed (control to PXF patients) ratio was planned to be 1:1 in this sample. Thus, the minimal sample size required for an 80% power and 5% significance assumption is 70 patients, 35 patients in each group.

Sample size was calculated using WinPepi software, version 11.65, 2016.

### Statistical analysis

Data were checked for normality using Shapiro Wilks test, Q–Q plot and histogram chart with a normal distribution curve. The student’s t test, the Mann–Whitney U test and the chi-square test, were used as appropriate to compare the baseline variables between the two groups. Overall differences between formula measurements for each group were described using non-parametric Friedman’s tests, with Bonferroni correction to reduce the risk of a type 1 statistical error in post-hoc pairwise comparisons. Indicators of measurements within ranges of ± 0.50/ ± 0.75/ ± 1.0 were compared pairwise between formulae using McNemar's tests with Holm-Bonferroni corrections.

The difference in absolute error between the PXF group and the control group for each formula were compared using a Mann–Whitney U test.

All hypothesis tests were two-sided and differences with *p* < 0.05 were considered statistically significant.

Statistical analysis was performed using IBM SPSS Statistics for Windows version 26.0 (IBM Corp., Armonk, NY, USA).

## Results

151 eyes of 151 patients (58% females) were included in this study. The mean age of patients was 73.9 ± 7.1 years (range 49–91 years). The post-operative BCVA was 0.12 ± 0.067 LogMAR in the PXF group and 0.11 ± 0.079 LogMAR in the control (*p* = 0.34). Post- operative SE in the PXF group was 1.12 ± 1.12 compared to -0.39 ± 0.18 (*p* = 0.025). Table 1 shows the demographic and clinical characteristics in the study groups.

**Table 1:** Demographic and clinical characteristics of the study groups

Demographic characteristics	PXF group (58 eyes)	Control group (93 eyes)	*P* value
Age, years	79.86 ± 7.32	77.22 ± 6.85	0.57
Gender (%)			
Female	31 (53%)	46 (53%)	0.92
Male	27 (47%)	44 (47%)	
Clinical characteristics			
AL (mm)	23.42 ± 1.11	23.37 ± 1.14	0.79
ACD (mm)	2.93 ± 0.43	3.03 ± 0.4	0.14
LT (mm)	4.72 ± 0.46	4.48 ± 0.55	0.23
WTW (mm)	11.78 ± 0.42	11.87 ± 0.47	0.27
MeanK	44.84 ± 1.63	44.49 ± 1.9	0.19
IOP (mmHg)	14.71 ± 3.05	14.06 ± 2.48	0.45
Glaucoma (%)	20.6%	4.3%	0.002
Post-operative BCVA (LogMAR), SD	0.12 ± 0.067	0.11 ± 0.079	0.34
Post-operative SE	1.12 ± 1.12	− 0.39 ± 0.18	0.025

The MAE for the BUII formula was 0.63 ± 0.87 D for the PXF group and 0.36D ± 0.48 for the control group (*p* = 0.04). The MAE for the Hill-RBF 3.0 formula was 0.61D ± 0.84 for the PXF group and 0.42D ± 0.55 for the control group (*p* = 0.05).

The standard deviation (once the mean error (ME) was optimized to zero) for the PXF group was 1.05D with the BUII formula and 1.02D with Hill-RBF 3.0 formula (*p* = 0.875). The standard deviation for the control group was 0.60D with the BUII formula and 0.69D with Hill-RBF 3.0 formula (*p* = 0.632).

The SRK/T formula had the highest MAE and MedAE and the lowest percentage of eyes achieving their refractive target of all formulae for both PXF group and control group.

The standard deviation (once the ME was optimized to zero) for the SRK/T formula was 1.07D for the PXF group and 0.68D for the control group (*p* = 0.04).

The MAE for the SRK/T formula was 0.68D ± 0.85 for the PXF group and 0.43D ± 0.53 for the control group (*p* < 0.05).

Friedman’s test (Table 2) shows MAE and MedAE in the PXF and control groups for the different formulae, as well as the comparison between PXF and controls and between the different formulae in each group. There were significant differences in MAE and MedAE between PXF group and control group for all the examined formulae (*p* < 0.05). In the PXF group there were no significant differences in MAE and MedAE between different formulae (*p* = 0.778). In the control group there were significant differences between in MAE and MedAE between different formulae (*p* < 0.001).

**Table 2:** formulae analysis

Formula	PXF group (58 eyes)			Control group (93 eyes)				
	MAE (SD)	MedAE (IQR)	p^*^	MAE (SD)	MedAE (IQR)	p^*^	p^*#^	p^**^
BUII	0.63 (0.87)	0.35 (0.11, 0.77)	0.778	0.36 (0.48)	0.25 (0.11, 0.43)	< 0.001		0.04
Hill-RBF 3.0	0.61 (0.84)	0.32 (0.10, 0.74)		0.42 (0.55)	0.30 (0.12, 0.50)		0.31 (Hill-RBF 3.0 VS BUII)	0.05
Haigis	0.64 (0.81)	0.40 (0.14, 0.79)		0.43 (0.45)	0.29 (0.17, 0.57)			0.03
Holladay I	0.64 (0.81)	0.35 (0.18, 0.69)		0.40 (0.46)	0.29 (0.18, 0.51)			0.01
Hoffer Q	0.62 (0.79)	0.42 (0.11, 0.72)		0.33 (0.46)	0.23 (0.10, 0.44)			0.05
SRK/T	0.68 (0.85)	0.41 (0.17, 0.85)		0.42 (0.53)	0.28 (0.14, 0.51)			0.04

MAE. Mean absolute error; MedAE. Median absolute error

p*Overall difference between formulae MedAE (Friedman’s test);

p*^#^Pairwise comparisons between formulae MedAE (with Bonferroni adjustment);

p**Difference within individual formula between PXF Group and Control Group (Wilcoxon signed rank test)

**Fig. 1:**
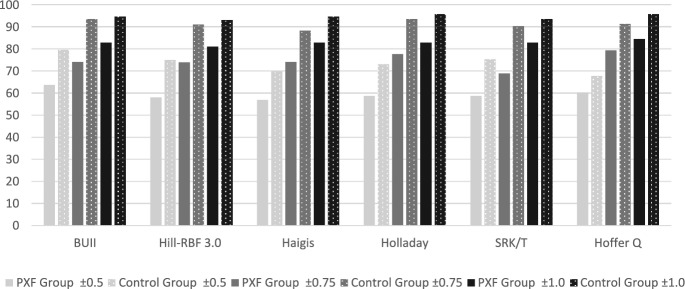
Proportion of eyes with absolute error in prediction below or equal to 0.50D, 0.75D, and 1.00D

Table 3 and Fig. 1 show the proportion of eyes that had an absolute error (AE) in prediction of 0.50 D or less, 0.75 D or less, and 1.00 D or less, for both PXF group and control group. The highest proportion of eyes achieving their refractive target within 0.5 D for both PXF group and control group were achieved by the BUII formula and the Hill-RBF 3.0. This difference is not preserved within 0.75 D and 1.0 D. (Table 3).

**Table 3:** Proportion of eyes with absolute error in prediction below or equal to 0.50 D, 0.75 D, and 1.00 D

Formula	PXF group						Control group					
	± 0.50 n (%)	*p*	± 0.75 n (%)	p	± 1.0 n (%)	*p*	± 0.50 n (%)	*p*	± 0.75 n (%)	*P*	± 1.0 n (%)	*P*
BUII	37 (63.79)		43(74.13)		48(82.75)		74 (79.57)		87 (93.54)		88 (94.62)	
Hill-RBF 3.0	34 (58.62)	0.412	43 (74.13)	0.674	47 (81.03)	0.681	70 (75.27)	0.470	85 (91.4)	0.389	87 (93.55)	0.307
Haigis	33 (56.89)	< 0.001	43 (74.13)	0.012	48 (82.75)	0.375	65 (69.89)	0.003	82 (88.17)	0.075	88 (94.62)	0.727
Holladay I	34 (58.62)	0.017	45 (77.58)	0.125	48 (82.75)	1.000	68 (73.11)	0.064	87 (93.54)	0.219	89 (95.69)	1.000
SRK/T	34 (58.62)	< 0.001	40 (68.96)	< 0.001	48 (82.75)	1.000	70 (75.25)	< 0.001	84 (90.32)	0.109	87 (93.54)	1.000
Hoffer Q	35 (60.34)	0.001	46 (79.31)	0.004	49 (84.48)	1.000	63 (67.74)	0.002	85 (91.39)	0.508	89 (95.69)	1.000

p Differences between proportions comparing formulae pairwise

## Discussion

Accuracy of IOL calculations and refractive outcomes of cataract surgery are an important concern for both surgeons and patients.

Previous studies addressed the subject of refractive outcomes in PXF patients, without evaluating formulae accuracy. Ishikawa et al.[13] reported a greater error in SE refraction in the PXF group than in the control group only in the very short post-operative period, 1 day post operatively, in both myopic and hyperopic directions. There was no significant difference between the groups in refractive outcomes at 1 month.

Manoharan et al. [14] investigated refractive outcomes of cataract surgery only in PXF glaucoma patients. In this research PXF glaucoma patients had an odds ratio of 7.3 of having a refractive surprise after phacoemulsification cataract surgery compared with controls. This research did not include patients with PXF syndrome without glaucoma, thus doesn’t represent the entire PXF patient’s population. In contrast to this study, our study includes both PXF patients with glaucoma and without glaucoma, making it more representative of PXF patient’s population in real life. Another study compared refractive precision of three-piece versus one-piece IOLs in eyes with PXF. Refractive precision in PXF eyes was found to be better with three-piece than with one-piece IOL implantation, but worse than with one-piece IOL implantation in non-PXF eyes [15]. Authors suggested that in patients with PXF haptic compression of the 3-piece IOL to the sclera restored the stability of the lens and prevented unexpected changes in ACD in PXF eyes that cause refractive errors.

Accuracy of PPOR is mainly affected by prediction of post-operative ELP [15, 16]. Studies have shown that errors in ELP prediction accounts for 22–38% of the total refractive prediction error [17, 18].

Fallah et al. [19] reported a deeper than excepted ACD postoperatively in eyes with PXF, representing a backward movement of the IOL affecting the predicted ELP. In another study, Güngör et al. [20] demonstrated that post-operative ACD change in patients with PXF syndrome is significantly greater compared with controls. Kassos et al. [21] also demonstrated ACD deepening as well as hyperopic shift following uneventful phacoemulsification surgery, attributed to zonular laxity and bag instability, affecting the ELP. Similarly, in our study we show a statistically significant hyperopic shift in the PXF group compared to a slight myopic shift in the control group. This could be explained by a posterior movement of the zonules in the PXF group post-operatively.

Another possible explanation for the inaccuracy of IOL calculations in patients with PXF in addition to ELP changes, could be post-operative IOL tilt and decentration, which is also attributed to zonular weakness and instability. A study that evaluated the minimum lens-iris distance (MLID) in eyes with PXF and age-matched controls, found that the MLID is lower in eyes with PXF [22]. Moreover, in this study MLID was measured in each quadrant of the iris and the differences inferior-superior and nasal-temporal were found to be different between PXF patients and controls, but not statistically significant. Quadrantwise differences in lens-iris distance could be related to crystalline lens position changes such as tilt and decentration in PXF patients. Although not statistically significant, these trends support our hypothesis that IOL tilt and decentration contribute to the inaccuracy in IOL calculations in PXF patients. The modern IOL calculation formulae are unable to predict such tilt or decentration, nor to adjust the result to such situations.

Once understanding the great effect that PXF has on ELP and the refractive outcome, it was incumbent to explore how this is reflected in the refractive results of the commonly used formulae today. In our study we investigated 151 eyes of 151 patients, divided in to 2 groups- PXF group (N = 58) and control group (N = 93).

The main finding in this study is the significant difference in accuracy of IOL calculation formulae between PXF group and control group. The MAE for the BUII and Hill-RBF 3.0 formulae was nearly doubled in the PXF group compared to the control group and was significantly different. (Table 2).

The standard deviation for the BUII and Hill-RBF 3.0 formulae was as well nearly doubled in the PXF group compared to the control group. (Table 2).

Higher percentage of eyes achieved refractive target in the control group compared to PXF group in all examined formulae (Table 3, Fig. 1). This finding is consistent with a recent study that showed lower accuracy of IOL calculations in patients with PXF syndrome compared to control [9]. That study’s population size was smaller than our study (N = 80, 42 patients in the PXF group and 38 patients in the control group) and examined only 3 formulae (Barrett Universal II (BUII), Hill-RBF 3.0 and SRK/T) while in our study we examined 6 formulae [9].

Another surprising finding in our study is that there was no significant difference in MAE and MedAE between all formulae in the PXF group. This means modern 4th generation formulae such as BUII and Hill RBF 3.0 are as accurate as 3rd generation formulae such as SRK/T when it comes to PXF eyes. These results can be explained by the limited ability to predict post-operative ELP in eyes with PXF. Another explanation might be that the zonular instability creates a slight tilt or decentration that is not clinically significant but is optically significant and influence the manifest refraction.

The percentage of eyes reaching refractive targets within 0.5 D or less, 0.75 D or less and 1.00 or less in the PXF group using all formulae is significantly lower than the control group. The highest proportions of eyes with AE of 0.50 D or less, 0.75 D or less, and 1.00 D or less occurred using the BUII formula (PXF group: 63.79%, 74.13%, and 82.75%, respectively. Control group: 79.57%, 93.54%, and 94.62%, respectively). Despite the ELP instability, the BUII and Hill-RBF 3.0 formulae provide more predictable and satisfactory results in PXF patients compared to the other formulae examined in this study. Hoffer Q is the only 3rd generation formula closest to the BUII in the PXF Group. An interesting finding which might be due to its unique calculation of ELP using the tangent of K.

The limitation of our study is the fact that the surgeries were conducted by 2 different surgeons and not a single surgeon, with possibly different ranges of proficiency and results, different surgically induced astigmatism etc. Both surgeons operated both patients in the PXF group and in the control group and had the same ratio of cases in both groups.

In conclusion, we found in this study significant difference in refractive outcomes between eyes with PXF and controls. There were significant differences in all the included formulae between PXF group and control group measures. Barret universal II formula had the highest proportion of eyes with absolute error in prediction below or equal to 0.50 D in both PXF and control groups.

Our main take home message for surgeons from this study is that PXF patients are prone to less accurate results, as well as often show hyperopic shift from predicted refraction, and should be informed preoperatively about it to avoid dissatisfaction.
